# Valproate, a Mood Stabilizer, Induces WFS1 Expression and Modulates Its Interaction with ER Stress Protein GRP94

**DOI:** 10.1371/journal.pone.0004134

**Published:** 2009-01-06

**Authors:** Chihiro Kakiuchi, Shinsuke Ishigaki, Christine M. Oslowski, Sonya G. Fonseca, Tadafumi Kato, Fumihiko Urano

**Affiliations:** 1 Program in Gene Function and Expression, University of Massachusetts Medical School, Worcester, Massachusetts, United States of America; 2 Program in Molecular Medicine, University of Massachusetts Medical School, Worcester, Massachusetts, United States of America; 3 RIKEN Brain Science Institute, Wako, Saitama, Japan; Victor Chang Cardiac Research Institute, Australia

## Abstract

**Background:**

Valproate is a standard treatment for bipolar disorder and a first-line mood stabilizer. The molecular mechanisms underlying its actions in bipolar disorder are unclear. It has been suggested that the action of valproate is linked to changes in gene expression and induction of endoplasmic reticulum (ER) stress-response proteins.

**Principal Findings:**

Here we show that valproate modulates the ER stress response through the regulation of WFS1, an important component for mitigating ER stress. Therapeutic concentrations of valproate induce expression of WFS1 mRNA and activate the WFS1 promoter. In addition, WFS1 forms a complex with GRP94, an ER stress-response protein, in which valproate dose-dependently enhances its dissociation from GRP94.

**Conclusions:**

These results suggest that the therapeutic effects of valproate in bipolar disorder may be mediated by WFS1 expression and its dissociation from GRP94.

## Introduction

Bipolar disorder is a severe mental disorder characterized by recurrent episodes of mania and depression, affecting about 0.5–1% of the population [1]. Although the pathogenesis of bipolar disorder is unclear, it is known that mood stabilizers, such as valproate, can prevent its recurrence [2]. Valproate, a simple branched-chain fatty acid, has been used in the treatment of bipolar disorder, epilepsy, and migraine [3], [4], [5]. Valproate increases the level of the inhibitory neurotransmitter γ-aminobutyric acid (GABA), with acute administration causing a 15%–45% increase in GABA in the brains of rodents. Because inhibition of GABAergic signaling can cause seizures and potentiation of GABA signaling can prevent seizures, this effect of valproate on GABA levels has been proposed as a mechanism for its anticonvulsant activity [3], [4], [5].

The molecular mechanisms of valproate in bipolar disorder are unclear. One hypothesis is that the therapeutic effect of valproate in bipolar disorder may be mediated by changes in expression of neuroprotective genes. Valproate increases the DNA binding of activator protein 1 (AP-1), a transcription factor which is a heterodimeric protein composed of proteins belonging to the c-Fos, c-Jun, and ATF families [6], [7]. This may lead to enhanced expression of AP-1 target genes that have important functions in neurons. In addition, valproate has been characterized as a histone deacetylases (HDAC) inhibitor and can regulate gene expression through epigenetic mechanisms [8]. These findings suggest an attractive possibility that valproate increases expression of multiple genes that have protective effects against bipolar disorder.

The unfolded protein response (UPR) is a gene expression program that modulates endoplasmic reticulum (ER) stress, a specific type of cell stress caused by the accumulation of misfolded proteins in the ER [9], [10]. GRP94 is a component of the UPR and has a function in protein folding and degradation [11], [12], [13]. Genetic variations in the GRP94 gene are associated with bipolar disorder in the Japanese population [14]. High throughput proteomics analysis revealed that GRP94 interacts with WFS1 protein (Fonseca and Urano, unpublished data). WFS1 is also a component of the UPR and regulates cellular ER stress levels [15]. WFS1 was initially identified as a causative gene for Wolfram syndrome, a rare autosomal recessive disorder characterized by diabetes insipidus, diabetes mellitus, optic atrophy and deafness [16], [17], [18]. About 60% of patients with Wolfram syndrome have some mental disturbance such as severe depression and psychosis [19]. Importantly, even the heterozygotes who do not have Wolfram syndrome are 26-fold more likely than non-carriers to have a psychiatric hospitalization [20], and the relative risk of psychiatric hospitalization for depression was estimated to be 7.1 [21]. These findings suggest that the modulation of ER stress by WFS1 and GRP94 may be involved in bipolar disorder.

Here we show that valproate regulates WFS1 and GRP94 in neurons. Valproate activates the WFS1 promoter and induces WFS1 mRNA expression in neuronal cells. Under normal conditions, WFS1 forms a complex with GRP94 and valproate enhances its dissociation from GRP94. Our data raise the possibility that the therapeutic effects of valproate in bipolar disorder may be mediated by the modulation of ER stress through the regulation of WFS1 and GRP94.

## Results

### Valproate increases the expression of WFS1 without inducing other ER stress markers

**Figure 1:**
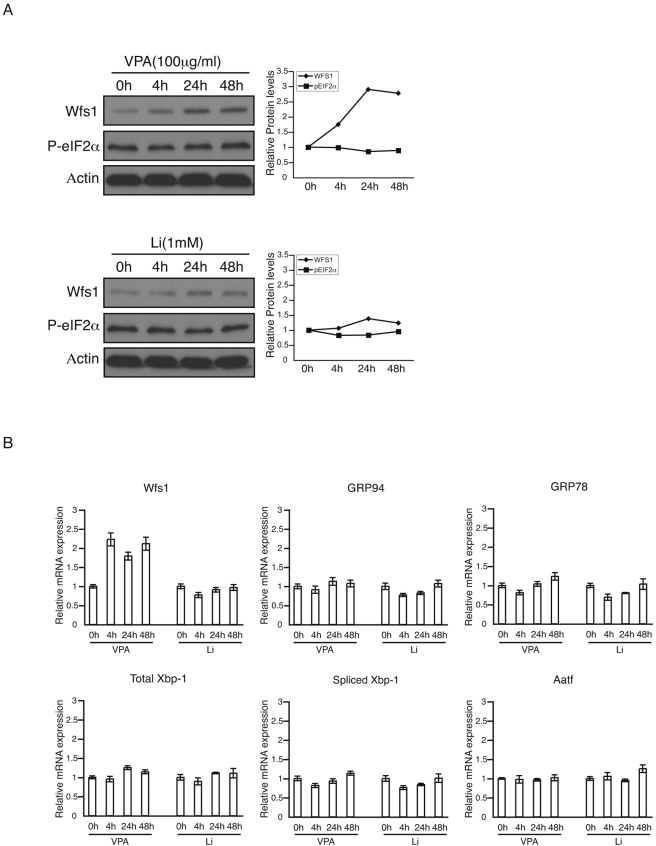
Valproate increases the expression of WFS1 without inducing other ER stress markers. (A) Neuro-2a cells were treated with valproate (VPA, 100ug/ml) or lithium (Li, 1mM) for 4 hr, 24 hr, and 48 hr. Expression levels of Wfs1, phospho-eIF2α (P-eIF2α) and Actin were measured by immunoblot. The relative amounts of the proteins, Wfs1 and P-eIF2α, which are adjusted by the amount of actin, are shown in the right panels. (B) Expression levels of Wfs1, GRP94, GRP78, total Xbp-1, spliced Xbp-1, and Aatf were measured by quantitative real-time PCR (n = 3; values are mean±SD).

To investigate the possible involvement of valproate in WFS1 function in neurons, we first determined expression levels of WFS1 in neuronal cell lines treated with a therapeutic concentration of valproate. Valproate increased WFS1 protein expression levels in Neuro-2a cells with a peak at 24 hr (Figure 1A, upper panel). Another mood stabilizer, lithium, did not increase WFS1 expression levels significantly in these cells (Figure 1A, lower panel).

WFS1 expression is regulated by the master regulators of ER stress signaling, PERK and IRE1α, under ER stress conditions [15]. To determine whether PERK signaling is involved in WFS1 upregulation by valproate, we measured expression levels of phosphorylated eIF2α, which reflect PERK activation levels. Valproate did not increase eIF2α phosphorylation levels (Figure 1A upper panel), raising the possibility that WFS1 upregulation by valproate is not regulated by the ER stress signaling network. To test this idea, we measured mRNA expression levels of common ER stress response genes, GRP94, GRP78, total and spliced XBP-1, and AATF by real-time PCR. Figure 1B shows that expression levels of these ER stress markers did not change by valproate, indicating that valproate specifically upregulates WFS1 without activating other components of ER stress signaling.

**Figure 2:**
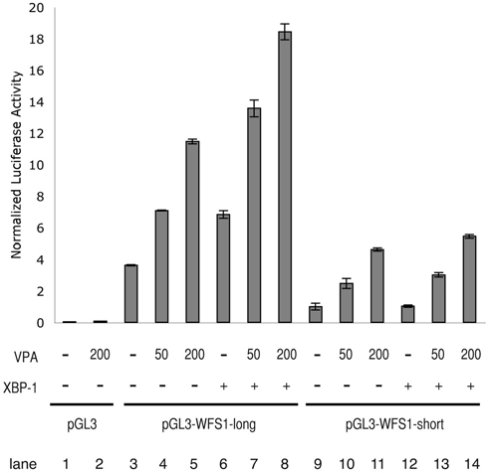
WFS1 promoter is activated by valproate. SH-SY5Y cells were transfected with a reporter plasmid containing 500 bases of the WFS1 promoter driving the luciferase gene (pGL3-WFS1-long), a control reporter plasmid containing only 60 bases of the WFS1 promoter (pGL3-WFS1-short), or control plasmid (pGL3) plus XBP-1 expression plasmid or control plasmid. The cells were then treated with two different concentrations of valproate, 50 µg/ml and 200 µg/ml, for 6 hr.

It has been proposed that WFS1 mRNA expression is regulated by a 500-base-pair promoter region located upstream of its transcriptional start site [22]. We were therefore interested in determining whether this WFS1 promoter can be activated by valproate treatment. We transfected a neuronal cell line, SH-SY5Y cells, with a reporter plasmid containing 500 bases of the WFS1 promoter driving the luciferase gene or a control reporter plasmid containing only 60 bases of the WFS1 promoter, then treated these cells with two different concentrations of valproate. Valproate led to a seven fold (50 µg/ml) and a twelve fold (200 µg/ml) induction of luciferase activity (Figure 2, lanes 4 and 5). The same promoter could not be activated in non-neuronal 293T cells (data not shown). It has been postulated that XBP-1 is important in activating the WFS1 promoter in SH-SY5Y cells [22]. We therefore considered the possibility that the addition of valproate to XBP-1 expression can enhance luciferease activity. To test this idea, we co-transfected SH-SY5Y cells with XBP-1 expression plasmid along with the WFS1 reporter plasmid or the control plasmid with or without valproate treatment. As we predicted, the addition of valproate enhanced the induction of luciferase activity by XBP-1 in a dose-dependent manner (Figure 2, lanes 7 and 8). Collectively, these results indicate that valproate can strongly activate the WFS1 promoter together with XBP-1 specifically in neuronal cells.

### Mood stabilizers modulate WFS1-GRP94 complex

**Figure 3:**
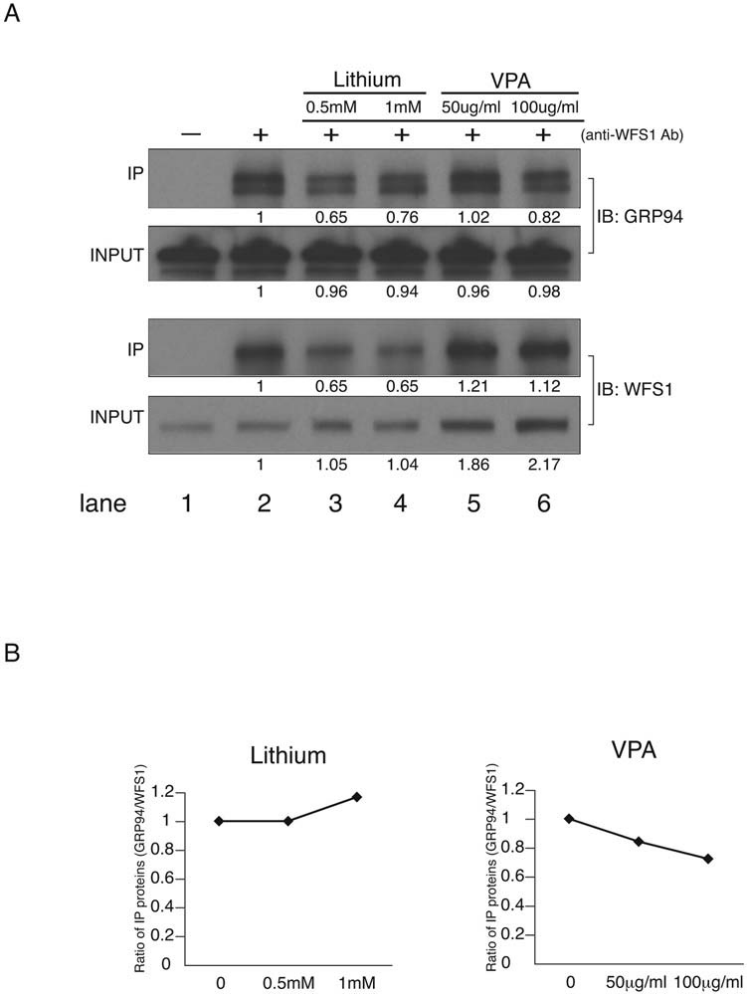
Mood stabilizers modulate the WFS1-GRP94 complex. (A) Neuro-2a cells were treated with lithium (Li, 0.5 mM, 1 mM), valproate (VPA, 50 ug/ml, 100 ug/ml) for 48 hr or untreated. Wfs1 was immunoprecipitated (IP) using lysates from the cells with anti-Wfs1 antibody. IP products were immunoblotted (IB) with anti-GRP94 antibody or anti-Wfs1 antibody. (B) The ratio of the relative amount of immunoprecipitaed GRP94 to that of immunoprecipitated WFS1 is shown. The X axis indicates the concentration of each drug.

High-throughput proteomics analysis has shown that WFS1 interacts with GRP94 (Fonseca and Urano, manuscript in preparation). To confirm this, we examined the association of WFS1 with GRP94 in Neuro-2a cells by immunoprecipitation. As we predicted, WFS1 associated with GRP94 under normal conditions (Figure 3A, lane 2, upper panels, IP).

It has been shown that valproate and lithium can modulate GRP94 expression in neurons [23], raising the possibility that this interaction can also be modulated with valproate and lithium. To test this possibility, we treated Neuro-2a cells with therapeutic concentrations of lithium or valproate for 48 hr, then examined the interaction between WFS1 and GRP94 by immunoprecipitation. The amount of GRP94 co-immunoprecipitated with WFS1 was decreased with lithium (Figure 3A, lanes 3 and 4, upper panels). The amount of WFS1 immnoprecipitated with anti-WFS1 antibody was also decreased with lithium (Figure 3A, lanes 3 and 4, lower panels, IP). Figure 3B shows that lithium treatment of Neuro-2a cells did not change the ratio between immunoprecipitated WFS1 and GRP94. These results suggest that lithium treatment may cause a conformational change of WFS1 protein, rendering the immunoprecipiation with anti-native-WFS1 antibody less efficient.

Valproate increased WFS1 expression levels in a dose-dependent manner (Figure 3A, lanes 5 and 6, lower panels, input). A parallel rise was observed in the amount of immunoprecipitated WFS1 (Figure 3A, lanes 5 and 6, lower panels, IP). Valproate decreased the ratio between immunoprecipiated WFS1 and GRP94 in a dose-dependent manner (Figure 3B), suggesting that WFS1 dissociates from GRP94 and that GRP94-free WFS1 is increased with valproate.

## Discussion

Although it is well established that valproate is a standard treatment for bipolar disorder and a first-line mood stabilizer, its mechanism of action has not been fully elucidated. Our results demonstrate that valproate induces expression of WFS1 and enhances its dissociation from GRP94 in neurons. We propose that the therapeutic effect of valproate is partially mediated by modulation of ER stress through the regulation of WFS1 and GRP94.

Valproate strongly activates the promoter region of WFS1 gene. We have previously shown that the minimum element for WFS1 promoter activation under ER stress conditions. The sequence of the element was similar to the one of ER stress response element (ERSE). We called it ERSE-like element [22]. The upregulation of WFS1 by valproate is probably regulated by the same element because the promoter lacking this sequence, pGL3-WFS1-short, did not respond to valproate (Figure 2). Consistent with previous results, this activation can be enhanced by co-transfection of the transcription factor XBP-1. It is possible that this activation might be indirect because our previous result indicated that XBP-1 could not directly bind to the ERSE-like element [22]. Other unknown transcription factors induced by XBP-1 or interacted with XBP-1 may have a function in the activation of WFS1 promoter.

**Figure 4:**
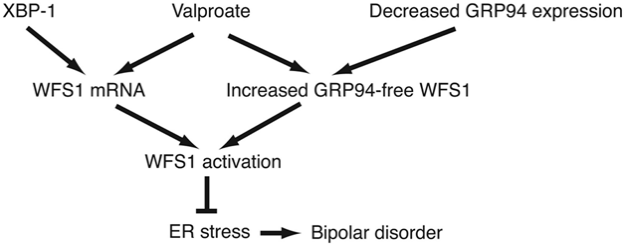
A speculative model of the action of valproate in the regulating of WFS1 and in the treatment of bipolar disorder.

High-throughput proteomics analysis revealed that GRP94 was one of the proteins that could interact with WFS1 in 293T cells (Fonseca and Urano, manuscript in preparation). Our data indicate that valproate enhances dissociation of WFS1 from GRP94. Considered collectively, valproate may regulate the UPR by modulating the interaction between GRP94 and WFS1. Genetic variations in the GRP94 gene are strongly associated with bipolar disorder in the Japanese population [14]. The protective GRP94 allele associated with bipolar disorder was related to low mRNA expression of GRP94 [14]. Downregulation of GRP94 may increase the amount of GRP94-free WFS1, leading to the enhancement of WFS1 function. Thus, the downregulation of GRP94 may have the same effect as the upregulation of WFS1. It is also possible that upregulation of WFS1 by valproate increases the ratio between GRP94-free WFS1 and GRP94-bound WFS1, leading to the activation of WFS1 (Figure 4).

GRP94 is an ER resident member of the HSP90 family of molecular chaperones. It has been shown that an HSP90 inhibitor, geldanamycin, can bind to GRP94, inhibit its function, and increase the transcription of ER molecular chaperones [24]. It would be possible that geldanamycin as well as its less toxic analogues, 17-AAG and GA, may synergize with valproate and increase its effect on WFS1 expression and modulation of the WFS1-GRP94 complex. Thus, inhibitors of GRP94 function could be a novel class of drug for bipolar disorder.

In this study, we focused on the function of valproate in WFS1 expression and its interaction with GRP94. The modulation of ER stress through the activation of WFS1 may be part of valproate's action in bipolar disorder. Our findings suggest that valproate and a compound that can reduce GRP94 expression in neurons may be a valuable treatment for patients with bipolar disorder.

## Methods

### Cell culture

Neuro-2a cells, SH-SY5Y, and 293T cells were maintained in DMEM with 10% fetal bovine serum.

### Immunoblotting and immunoprecipitation

Cell extracts were prepared by lysis in TNE buffer (50 mM Tris-HCl pH7.5, 150 mM NaCl, 1 mM EDTA and 0.1% NP40) containing protease inhibitors and phosphatase inhibitor Cocktail 2 (SIGMA) for 15 min on ice, then the extracts were cleared by centrifuging at 12,000 g for 20 min at 4°C. Extracts were normalized for total protein (10 µg per lane), separated using 4%–20% linear gradient SDS-PAGE (Bio Rad, Hercules, CA) and electroblotted. Blots were probed with the following antibodies: anti-actin (Sigma, St. Louis, MO); anti-phospho-eIF2α, anti-GRP94 (Cell Signaling, Danvers, MA). The amount of protein was quantified using ImageJ software. For the immunoprecipitation, cells extracts were prepared by lysis in TNE buffer containing protease inhibitors for 15 min on ice. WFS1 was immunoprecipitated from the extracts with anti-WFS1 antibody, a gift from Drs. Hisamitsu Ishihara and Yoshitomo Oka (Tohoku University, Japan).

### Luciferase Assay

SH-SY5Y cells were transfected with a reporter plasmid containing 500 bases of the WFS1 promoter driving the luciferase gene (pGL3-WFS1-long), a control reporter plasmid containing only 60 bases of the WFS1 promoter (pGL3-WFS1-short), or control plasmid (pGL3) plus XBP-1 expression plasmid or control plasmid using Lipofectamine™ 2000 (Invitrogen, Carlsbad, CA). 48 hrs post-transfection, the cells were treated with two different concentrations of valproate, 50 µg/ml and 200 µg/ml, for 6 hr and then lysed using a Luciferase Assay System kit (Promega, Madison, WI). The light produced from the samples was read by a plate reading luminometer, Victor X (Perkin Elmer, Waltham, MA). Each sample was read in triplicate and normalized against the signal produced from mock wells.

### Real-time polymerase chain reaction

Total RNA was isolated from the cells with the RNeasy Mini Kit (Qiagen, Valencia, CA) and reverse transcribed using 1 µg of total RNA from cells with Oligo-dT primer. For the thermal cycle reaction, the iQ5 system (BioRad, Hercules, CA) was used at 95°C for 10 min, then 40 cycles at 95°C for 10 sec, and at 55°C for 30 sec. The relative amount for each transcript was calculated by a standard curve of cycle thresholds for serial dilutions of cDNA sample and normalized to the amount of actin. The polymerase chain reaction (PCR) was done in triplicate for each sample, then all experiments were repeated three times. The following sets of primers and Power SYBR Green PCR Master Mix (Applied Biosystems, Foster City, CA) were used for real-time PCR: for mouse actin, GCAAGTGCTTCTAGGCGGAC and AAGAAAGGGTGTAAAACGCAGC; for mouse WFS1, CCATCAACATGCTCCCGTTC and GGGTAGGCCTCGCCATACA; for mouse GRP94, AAGAATGAAGGAAAAACAGGACAAAA and CAAATGGAGAAGATTCCGCC; for mouse GRP78, TTCAGCCAATTATCAGCAAACTCT and TTTTCTGATGTATCCTCTTCACCAGT; for mouse total XBP-1, TGGCCGGGTCTGCTGAGTCCG and GTCCATGGGAAGATGTTCTGG; for mouse spliced XBP-1, CTGAGTCCGAATCAGGTGCAG and GTCCATGGGAAATGTTCTGG; for mouse AATF, TTCTTGGCAAACCGGAGC and AGCGTCTCTGGTTCTCCTGG.
